# Therapy of newly diagnosed follicular lymphoma

**DOI:** 10.3389/fonc.2012.00188

**Published:** 2012-12-11

**Authors:** Jason R. Westin, Sattva S. Neelapu

**Affiliations:** University of Texas MD Anderson Cancer CenterHouston, TX, USA

**Keywords:** newly diagnosed, B cell, lymphoma, follicular lymphoma, observation, immunotherapy, chemotherapy, outcomes

## Abstract

Newly diagnosed follicular lymphoma is relatively common and can be effectively treated with several differing approaches. Although the disease is often considered incurable, it is highly responsive to therapy when indicated. This review discusses the indications for treatment, risk stratification systems, treatment options with supporting clinical trial data, and expected therapeutic outcomes in newly diagnosed follicular lymphoma.

## INTRODUCTION

Follicular lymphoma is the second most common subtype of lymphoma with nearly 15,000 new cases annually in the United States (Siegel et al., 2011). For unclear reasons, the incidence of FL continues to increase in the United States and Europe (Cartwright et al., 1999; Groves et al., 2000). FL arises from germinal-center B cells with the genetic hallmark of an acquired t(14;18) (q32;q21) translocation, leading to deregulation of BCL2, a key gene in the regulation of apoptosis and cell death. The t(14;18) translocation is found in the normal B cells of over half of healthy individuals, and thus is insufficient alone for lymphomagenesis (Staudt, 2007). Occasionally, an *in situ* type of FL is identified with t(14;18) and BCL2 expression in a lymph node without other sites of disease. Patients with *in situ* FL rarely progress to disseminated FL and generally can be observed (Limpens et al., 1991; Cong et al., 2002; Roulland et al., 2006; Staudt, 2007; Jaffe, 2009). Historically, cells harboring the t(14;18) translocation in the peripheral blood of healthy individuals were thought to represent naïve B cells, but recent work suggests these cells have many similarities to FL cells, including class-switch recombination, and surface expression of IgM and IgD (Roulland et al., 2006). It is not clear if the presence of the t(14;18) translocation in healthy individuals has any prognostic or therapeutic value, but suggests a possible common pre-malignant stage.

## PROGNOSTIC SYSTEMS

Nearly five of every six patients with newly diagnosed FL will have advanced disease, either due to a long asymptomatic phase prior to diagnosis or early dissemination. Follicular lymphoma is staged by the Ann Arbor system, originally devised in the 1970s to account for radiation field size in Hodgkin lymphoma. The system accounts for a stepwise progression to adjacent lymph node chains, a pattern often not found in non-Hodgkin lymphoma (NHL), and thus its validity in this population has been questioned (Staudt and Dave, 2005). Clinical outcomes vary within stages, suggesting that clinical factors other than location of disease affect outcome.

**FIGURE 1:**
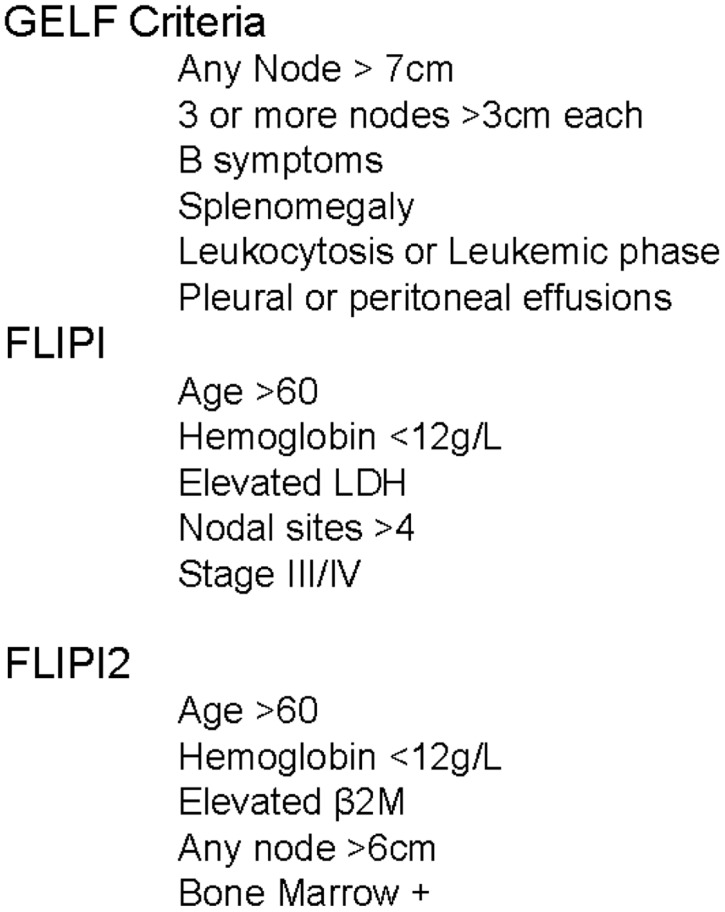
**Follicular lymphoma therapy indications and clinical predictors**.

In 2000, the Intergruppo Italiano Linfomi attempted to account for these additional factors by proposing a model incorporating age, sex, number of extranodal sites of disease, B symptoms, serum lactate dehydrogenase (LDH), and erythrocyte sedimentation rate (ESR; Federico et al., 2000). This model was further improved by an international cooperative effort evaluating over 4000 patients, creating the Follicular Lymphoma International Prognostic Index (FLIPI, **Figure 1**). Based on the number of adverse features present, patients were classified into three groups with 10-year overall survival (OS) rates of 70.7, 50.9, and 35.5% (Solal-Céligny et al., 2004). The FLIPI was widely adopted, but had several important limitations. It was created from a retrospective database analysis of patients not treated with current chemoimmunotherapy, was missing significant amounts of data (ESR, β2M, and performance status), and had an endpoint of OS. OS is difficult to model in an indolent disease such as FL due to the duration of follow-up required, a relapsing and remitting disease course, and multiple lines of effective therapy. To account for these weaknesses, the International Follicular Lymphoma Prognostic Factor Project launched the F2 study in 2003 (Federico et al., 2009). The primary endpoint of the model was progression-free survival (PFS), now the preferred metric in lymphoma clinical trials (Cheson et al., 2007). The factors identified as significant in the F2 study, now referred to as the FLIPI2, are relatively similar to those included in the FLIPI score (**Figure 1**). Based on the number of factors present, the FLIPI2 classifies patients into three groups with 5-year PFS of 79.5, 51.2, and 18.8%.

It is important to note that the vast majority of patient data utilized to generate these models is from patients with grades 1 and 2 disease. Grade 3 FL is split into A and B categories by the WHO classification. The B category lacks centrocytes and has similar biology and outcomes to diffuse large B cell lymphoma (DLBCL; Jaffe, 2009). Grade 3B FL is generally treated like DLBCL, however this remains an area of controversy. The discussion in this paper refers to FL grades 1–A, unless otherwise stated.

As the majority of patients have a responding and relapsing course, the option of not treating immediately after diagnosis, often referred to as “watch and wait,” was explored and found to have no detrimental effect on OS (Horning and Rosenberg, 1984; O’Brien et al., 1991). More concerning, early retrospective reviews found that multiple single agents and chemotherapy combinations, though initially effective, had little or no effect on the OS of patients with FL (Horning, 1993). Newer studies which include therapy with targeted agents have demonstrated improvements in survival; however cure remains elusive for the vast majority of FL patients. Thus, newer treatments which prolong remission duration with minimal toxicity are needed.

## INDICATIONS FOR THERAPY

Most cases of FL demonstrate an indolent nature, and when treated are highly responsive to therapy (Cheson, 2010). Nearly all advanced FL will eventually relapse after response to initial therapy, and thus FL is currently considered incurable in most cases.

After appropriately staging a newly diagnosed patient with FL, the most important initial decision is “Does this patient require therapy now?” To assist in answering this question, several risk stratification classification systems have been generated for FL. The Groupe d’Etude des Lymphomes Folliculaires (GELF) criteria (**Figure 1**) were developed in a prospective clinical trial randomizing patients to observation, chemotherapy, or interferon (Brice et al., 1997). In this trial, patients with a high tumor burden had a significantly worse OS (5-year: 78 vs. 57%). Based on these data, patients with ≥3 nodal sites with diameters of ≥3 cm or one mass ≥7 cm are typically not candidates for delayed therapy.

If a patient does not require immediate treatment based on the above criteria, the options include “watch and wait,” immunotherapy with or without chemotherapy, and or radiotherapy.

## WATCH AND WAIT

Deferred therapy with serial observation, or “watch and wait,” has been employed in asymptomatic patients with low disease burden due to the typically indolent nature of FL, occasionally even demonstrating regression or long periods of disease stability without therapy (Horning and Rosenberg, 1984). Spontaneous regressions, occurring in 5–25% patients, are thought to be related to the immune responsive nature of FL (Kannan and Neelapu, 2009). In the GELF-86 trial, patients with newly diagnosed FL were randomized to receive interferon, the oral alkylating agent prednimustine, or observation until clinically meaningful progression (Brice et al., 1997). The overall response rate (ORR) to therapy was 70 and 78% in the early treatment arms, and 70% once therapy was indicated in the observation arm, and OS at 5 years was similar in all three arms. In another immediate vs. delayed therapy clinical trial, nearly 10% of the patients randomized to the observation arm did not require systemic therapy with at least 10 years of follow-up (Ardeshna et al., 2003). Several other trials have demonstrated similar outcomes with initial and delayed therapy (Abramson et al., 2009; Kloo et al., 2011). Even with modern therapies yielding improved survival, there are no conclusive data yet which suggest “watch and wait” results in inferior long-term survival outcomes.

The risk of transformation to a more aggressive form of lymphoma is a dreaded event in FL patients, as even intensification of therapy typically results in poor outcomes with the majority of patients dying within 2 years (Yuen et al., 1995). A large retrospective observational study found the annual risk of transformation to be an estimated 3% per year in patients initially treated with chemotherapy, radiotherapy, or observation (Al-Tourah et al., 2008).

An important factor to consider with the “watch and wait” strategy is patient anxiety. It is certainly understandable that some patients may find it difficult to receive a diagnosis of cancer and be given a follow-up visit in lieu of cancer therapy. Patient anxiety is often lessened when hearing the rationale behind “watch and wait,” and the advantages of deferring exposure to potential treatment-associated toxicities.

## RADIOTHERAPY

Limited stage FL (stage I and contiguous stage II) is potentially curable with radiotherapy alone. Several series have reported dramatic long-term disease-free survival (35–50% at ≥10 years, with 100% local control in the radiation field), likely representing cure, in patients who were treated with involved field radiotherapy with minimal long-term toxicities (Mac Manus and Hoppe, 1996; Wilder et al., 2001). A large trial of aggressive chemotherapy (prior to rituximab) with 30–40 Gy involved field radiotherapy resulted in a 10-year time to treatment failure and OS rates of 72 and 80%, respectively (Seymour et al., 2003). A randomized trial is currently evaluating radiotherapy with and without chemotherapy in this population. As neither chemotherapy nor immunotherapy are currently able to achieve this degree of long-term disease control, radiotherapy is considered the standard of care and should be strongly considered in this patient population.

## IMMUNOTHERAPY

The immune responsive nature of certain malignancies, including FL, has been successfully manipulated for therapeutic benefit using multiple strategies. In the late ninetieth century, Dr. William Coley used a mixture of bacterial toxins as an initial attempt at immunotherapy and reportedly achieved dramatic results in lymphomas. Unfortunately, these results could not be replicated (Thotathil and Jameson, 2007). The immune stimulant interferon-α demonstrated significant activity in follicular lymphoma, either in combination with chemotherapy or alone, but did not have a clear impact on OS and is not commonly utilized due to treatment related toxicities (Price et al., 1991; Solal-Celigny et al., 1993; Solal-Céligny et al., 1998; Herold et al., 2007; Baldo et al., 2010).

Dr. Levy and others proposed using monoclonal antibodies, a passive immunization strategy, to target lymphoma cell surface markers. In 1994, the phase I trial of a monoclonal antibody targeting the B cell marker CD20 (IDEC-C2B8, rituximab) displayed tumor regression in 40% of heavily pre-treated B cell lymphoma patients (Maloney et al., 1994). The mechanism of action of rituximab is thought to be a combination of complement-mediated cytotoxicity, antibody-dependent cellular cytotoxicity, and direct signaling. Rituximab has shown significant single agent activity in both the frontline and relapse settings (**Table 1**; Maloney et al., 1997; McLaughlin et al., 1998; Witzig et al., 2005). Importantly, these multicenter trials also found rituximab to be well tolerated with major toxicities essentially limited to infusional reactions.

**Table 1:** Clinical outcome with various therapeutic regimens in newly diagnosed FL (top) and promising experimental therapies in development in FL (bottom).

	ORR	CR	PFS (month)	Reference
**Rituximab**				
Previously treated	46–48	6–8	10–13	Maloney et al. (1997), McLaughlin et al. (1998)
Untreated	72	36	26	Witzig et al. (2005)
R-CHOP	96	20	NR, 30 months = 75%	Hiddemann et al. (2005)
CHOP	90	17	30 months	Hiddemann et al. (2005)
BR	92.7	39.8	69.5	Rummel et al. (2012)
R-CHOP	91.3	30	31.2	Rummel et al. (2012)
R-CHOP	85	41	NR, 2 years = 76%	Press et al. (2011)
CHOP-RIT	83	46	NR, 2 years = 80%	Press et al. (2011)
R-lenalidomide	98	87	NR, 14 months = 94%	Fowler et al. (2011)
**Experimental therapies**	**Target**	**Current development**		**Reference**
Ibrutinib	BTK	Phase II		Advani et al. (2012)
CAL 101	P13K	Phase II		de Vos et al. (2011)
ABT-263	Bcl2	Phase I/II		Wilson et al. (2009)
Epratuzumab	CD22	Phase II		Leonard et al. (2008)
Inotuzumab	CD22	Phase III		Ogura et al. (2010)
SAR3419	CD19	Phase I		Younes et al. (2009)
SGN-40	CD40	Phase I		Advani et al. (2006)
Blinatumomab	CD19/T cell	Phase I		Viardot et al. (2010)
Pidilizumab	PD-1	Phase II		Westin et al. (2010)
PF-05082566	4-1BB	Phase I		Fisher et al. (2012)
BiovaxID	Idiotype	Phase III		Schuster et al. (2011)****

*ORR, overall response rate; CR, complete response; PFS, progression free survival; RIT, radioimmunotherapy.*

In an attempt to directly address “watch and wait” vs. immediate immunotherapy, an intergroup trial randomized newly diagnosed FL patients with stage ≥II, asymptomatic non-bulky disease to observation, weekly rituximab × 4 doses, or weekly rituximab × 4 doses followed by maintenance rituximab (MR) every 2 months for 2 years (Ardeshna et al., 2010). The primary endpoints were time to initiation of new therapy and quality of life. Preliminary results show the estimated median time to initiation of new therapy was 33 months in the observation arm and not reached at 4 years in the rituximab arms. Perhaps not surprisingly, no difference in OS was found between the groups. It remains unclear if the delay in time to initiation of new therapy achieved by initial rituximab will ultimately change the natural history of the disease, future responses to rituximab, or time to second line therapy.

The PRIMA study demonstrated the value of continued exposure to rituximab in a less frequent schedule, commonly referred to as maintenance therapy (Salles et al., 2011). Over a thousand patients with newly diagnosed FL who achieved CR or PR after chemoimmunotherapy were randomized to observation or rituximab once every 2 months. At 2 years, 75% of patients treated with rituximab maintenance were free from progression, as opposed to 58% of observation patients (*p* < 0.0001). The RESORT trial randomized newly diagnosed FL patients who responded to rituximab weekly × 4 to receive indefinite MR or rituximab retreatment (RR) at the time of disease progression (Kahl et al., 2011). The median time to treatment failure was 3.9 years with MR and 3.6 with RR, and 95% of MR and 86% of RR did not require cytotoxic chemotherapy at 3 years. The mean number of rituximab doses were quite different: the MR group received 15.8 (range 5–31) and RR 4.5 (range 4–16) doses, raising the question of the cost effectiveness of the maintenance approach. No difference in OS was observed in either the PRIMA or RESORT trials.

Lenalidomide, an immunomodulatory agent that is FDA approved to treat multiple myeloma, has recently shown very promising efficacy in both newly diagnosed and relapsed FL (Witzig et al., 2009; Fowler et al., 2010; Zhu et al., 2011; Leonard et al., 2012). A phase II study of lenalidomide with rituximab in newly diagnosed FL patients revealed an impressive preliminary ORR of 98% and CR of 87% (Fowler et al., 2010, 2011). An international randomized trial comparing rituximab with either lenalidomide or chemotherapy in newly diagnosed FL is currently underway.

## CHEMOTHERAPY

Follicular lymphoma is a chemosensitive disease, with numerous combinations successfully utilized over the past 30 years. A retrospective review spanning 25 years from our institution found that with each subsequent intensification of initial therapy, both failure-free survival (FFS) and OS improved (Liu et al., 2006). The 1972–1982 cohort was treated with cyclophosphamide, doxorubicin, vincristine, prednisone (CHOP), and bleomycin, which resulted in a 15-year FFS and OS of 13 and 27%, respectively. In contrast, the 1988–1992 cohort was treated with intensive alternating triple therapy and interferon, and achieved a 15-year FFS and OS of 32 and 50%, respectively. Newer therapies, including those with rituximab, had not yet reached their median FFS and OS at the time of publication but appeared to result in further improvements. As with any study measuring long-term outcomes over different eras, a caveat is required for the possible influence of improved supportive care and subsequent therapies on survival.

The addition of rituximab to less intensive chemotherapy regimens, including CHOP and CVP, has proven in multiple studies to be superior to chemotherapy alone (Czuczman et al., 2004; Marcus et al., 2008). A randomized trial by the German Low-Grade Lymphoma Study Group of CHOP with or without rituximab found superior response rates, duration of response, and OS in the rituximab arm (**Table 1**; Hiddemann et al., 2005). As a result, rituximab is now established as a core component of nearly every therapy for FL, both as a single agent and combined with chemotherapy.

The combination of rituximab and bendamustine, an unique drug with activity similar to alkylating agents and purine analogs, has demonstrated significant activity in untreated and relapsed FL (**Table 1**; Rummel et al., 2005; Cheson and Rummel, 2009). A randomized study of rituximab combined with bendamustine or CHOP in newly diagnosed FL reported a superior PFS of 69.5 vs. 31.2 months favoring bendamustine (Rummel et al., 2012). Although the final report of this trial has not been published to date, the combination of bendamustine and rituximab is now commonly used for newly diagnosed patients requiring therapy.

## RADIOIMMUNOTHERAPY

In patients with newly diagnosed advanced stage FL treated with CHOP chemotherapy (without rituximab), a single dose of ^131^I-tositumomab resulted in an ORR of 91% and CR of 69% (Press et al., 2006). A randomized phase III study of R-CHOP vs. CHOP followed by ^131^I-tositumomab achieved similar results (2-year PFS of 76 and 80%, OS 97 and 93%, respectively; Press et al., 2011). An ongoing trial of R-CHOP followed by radioimmunotherapy consolidation and MR aims to determine if efficacy can be further improved by combining multiple anti-CD20 therapeutic modalities. A single course of ^131^I-tositumomab in newly diagnosed FL without chemotherapy resulted in an ORR of 95%, CR of 75%, and a median PFS of 6.1 years, an approach which may be valuable to elderly patients who fail rituximab monotherapy and decline chemotherapy (Kaminski et al., 2005).

## POST-THERAPY FOLLOW-UP

According to the NCCN guidelines, patients with initially treated FL should be followed with physical exam and laboratory assessment every 3–6 months for at least 5 years. Surveillance CT scans should be performed no more than every 6 months for the first 2 years, then annually if indicated. Patients and physicians may feel more comfortable with frequent imaging, but the associated cost and radiation exposure are not likely more advantageous than early diagnosis of relapse in this indolent disease. When relapse is identified, immediate therapy is not required and the same general criteria used in newly diagnosed patients can aide in determining when to start therapy.

If a patient appears to have relapsing disease with a rapidly enlarging site, significantly elevated LDH, or new B symptoms, it is possible they have transformed into an aggressive lymphoma (Wong and Dickinson, 2012). In this setting, it is imperative to obtain a biopsy of the concerning site, as the most accessible lymph node may still represent low-grade FL. Patients with FL which has undergone transformation have a poor prognosis and should be considered for a clinical trial.

## OUTCOMES

The National LymphoCare Study evaluated treatment practices of academic and community oncologists and found that 17% opted for initial observation, 13.9% rituximab monotherapy, 51.9% rituximab with chemotherapy, 5.6% radiation therapy, and only 6.1% on a clinical trial. These data were obtained prior to the data regarding bendamustine, and thus the majority of chemotherapy-treated patients received R-CHOP. With the perception that bendamustine has less toxicity than CHOP, these data have likely shifted to include more use of chemotherapy overall, with CHOP making up a smaller proportion. Of note, only 23% of stage I patients received radiation therapy – a potentially curative approach (see Radiation).

Newly diagnosed FL patients often are confused regarding their long-term prognosis, as FL is often considered “incurable.” It is important to clarify the important difference between incurable and untreatable. Patients may read information on the Internet and not understand this difference, and thus be unnecessarily concerned or question the recommendations of their doctor. The median OS for all newly diagnosed FL patients is >10 years, and these data are not likely representative of modern therapy as they are based on patients diagnosed in the pre-rituximab era (Liu et al., 2006).

## INVESTIGATIONAL APPROACHES

The LymphoCare study found that only 6.1% of patients were enrolled on a clinical trial for newly diagnosed FL. This may be due in part to the perception that FL is easily treated with standard therapies that achieve satisfactory outcomes. Although we agree with these assumptions, we recommend all newly diagnosed patients with FL be considered for clinical trial enrollment. Follicular lymphoma remains an incurable disease that requires costly, and occasionally toxic, recurrent treatments. An impressive assortment of new targeted agents and immunotherapies are undergoing clinical evaluation (**Table 1**; Advani et al., 2006, 2012; Leonard et al., 2008; Wilson et al., 2009; Younes et al., 2009; Ogura et al., 2010; Viardot et al., 2010; Westin et al., 2010; de Vos et al., 2011; Schuster et al., 2011; Fisher et al., 2012). The ultimate determination if these potential advances can improve outcomes for patients with FL depends upon having a significant number of both newly diagnosed and relapsed patients enroll on clinical trials.

## CONCLUSION

Follicular lymphoma is a common disease due to its incidence and relatively long life expectancy. Despite it being currently considered incurable, many effective treatments exist with many more under evaluation. Patients with early stage disease should be treated with radiotherapy, which is potentially curative. Newly diagnosed advanced stage disease patients should not reflexively be treated unless they are symptomatic or meet standardized criteria. When treatment is necessary, immunotherapy or immunochemotherapy are very effective, but eventual relapse is common. We recommend all newly diagnosed FL patients be considered for a clinical trial to accelerate the development of the next generation of therapy.

## Conflict of Interest Statement

The authors declare that the research was conducted in the absence of any commercial or financial relationships that could be construed as a potential conflict of interest.
